# Prefrontal cortex hemodynamic activity during a test of lower extremity functional muscle strength in children with cerebral palsy: A functional near-infrared spectroscopy study

**DOI:** 10.1111/ejn.16211

**Published:** 2023-12-21

**Authors:** Joel Licea, Owais A. Khan, Tarkeshwar Singh, Christopher M. Modlesky

**Affiliations:** 1 Department of Kinesiology, University of Georgia, Athens, GA, USA; 2 Department of Kinesiology, The Pennsylvania State University, State College, PA, USA

**Keywords:** brain activity, fNIRS, functional neuroimaging, motor control, muscle strength, neurorehabilitation, pediatrics

## Abstract

Children with cerebral palsy (CP) exhibit impaired motor control and significant muscle weakness due to a brain lesion. However, studies that assess the relationship between brain activity and performance on dynamic functional muscle strength assessments in CP are needed. The aim of this study was to determine the effect of a progressive lateral step-up test on prefrontal cortex (PFC) hemodynamic activity in children with CP. Fourteen ambulatory children with spastic CP (Gross Motor Function Classification System level I; 5–11 y) and 14 age- and sex-matched typically developing control children completed a progressive lateral step-up test at incremental step heights (0, 10, 15 and 20 cm) using their non-dominant lower limb. Hemodynamic activity in the PFC was assessed using non-invasive, portable functional neuroimaging (functional near-infrared spectroscopy). Children with CP completed fewer repetitions at each step height and exhibited lower PFC hemodynamic activity across step heights compared to controls. Lower PFC activation in CP was maintained after statistically controlling for the number of repetitions completed at each step height. PFC hemodynamic activity was not associated with LSUT task performance in children with CP, but a positive relationship was observed in controls at the most challenging 20 cm step height. The results suggest there is an altered PFC recruitment pattern in children with CP during a highly dynamic test of functional strength. Further studies are needed to explore the mechanisms underlying the suppressed PFC activation observed in children with CP compared to typically developing children.

## 1 | INTRODUCTION

Cerebral palsy (CP) is the most common cause of motor disability in childhood (Kirby et al., 2011). It arises from a non-progressive lesion in the developing brain. Individuals with CP exhibit impaired neuromuscular function and strength deficits (Mockford & Caulton, 2010). Muscle weakness is a major contributor to the lower aerobic capacity (Verschuren & Takken, 2010), low physical activity levels and functional mobility deficits exhibited by children with CP (Ferland et al., 2012). Secondary manifestations of CP include the underdevelopment of the musculoskeletal system (Modlesky et al., 2008), and increased risk of cardiometabolic disease (Batson et al., 2023; Peterson et al., 2015). The progressive lateral step-up test (LSUT) is a novel assessment of lower body functional strength that incorporates multiple step heights, with a graded increase in cardiometabolic and energy requirements placing higher attention and planning demands on the neuromotor system. Performance on the progressive LSUT requires the optimal allocation of metabolic resources, sustained attention to ensure consistent effort across increasing step heights, and appropriate emotional responses to fatigue.

An important brain region that mediates the functions required to perform the progressive LSUT is the prefrontal cortex (PFC). In addition to regulating the distribution of the brain’s limited metabolic resources (Alonso et al., 2013), the PFC also mediates executive functions like action planning, sustained attention and decision-making that are essential for successful goal-directed movements (Miller & Cohen, 2001). While not directly involved in motor command execution, the PFC has extensive connections with cortical and subcortical motor areas (Middleton & Strick, 2002) that allow it to exert significant influence over motor performance (Bigliassi & Filho, 2022). The PFC plays an important role in regulating both the rate and consistency of motor output, as well as modulating the emotional responses to perceived movement intensity and effort (Perrey & Besson, 2018). Given the metabolic, sensorimotor and attentional deficits exhibited by children with CP (Bottcher et al., 2010; Short et al., 2020), examining the role of the PFC in driving motor performance in children with CP is important.

Functional near-infrared spectroscopy (fNIRS) is a functional neuroimaging tool that yields results similar to those of functional magnetic resonance imaging (MRI) (Huppert et al., 2006), but without many of the limitations, such as susceptibility to movement artefacts, and inability to perform brain imaging during highly dynamic mobile tasks like the progressive LSUT inside the MRI scanner (Herold et al., 2017). fNIRS captures both task-evoked hemodynamic changes in the outer 3–5 mm of cortical tissues (Perrey, 2008) and hemodynamic changes in superficial tissues that constitute physiological noise of both local and global origin that must be accounted for during data processing (Obrig & Villringer, 2003). fNIRS provides higher temporal resolution (12.5 Hz, typically) compared to functional MRI (0.3–0.6 Hz) and improved identification of signal contaminants like respiration changes (~0.2–0.3 Hz) and cardiac pulsations (~1 Hz) (Tong et al., 2011). These factors make fNIRS a viable neuroimaging option for dynamic tasks like the progressive LSUT (Pinti et al., 2018).

Despite these advantages, few studies have assessed PFC hemodynamic activity during motor tasks in children with CP. Studies in other neurologically impaired populations reported increased PFC activation during gait and postural control tasks in individuals with Parkinson’s disease and post-stroke (Gramigna et al., 2017; Stuart et al., 2018). The sole study assessing PFC hemodynamic activity during a functional mobility task in children with CP reported decreased PFC activity during a robot-assisted walking task, with increased PFC activity observed post-training associated with improvements in gait characteristics (Perpetuini et al., 2022). These observations support the potential of PFC hemodynamic activity levels to serve as possible biomarkers of functional recovery following brain injury (Stinear, 2017; Surkar et al., 2018).

Exploring the role of the PFC during a progressive LSUT could shed light on mechanisms contributing to functional muscle strength deficits in children with CP. This study aimed to determine if PFC hemodynamic activity differs between children with CP and typically developing children during a progressive LSUT.

## 2 | MATERIALS & METHODS

### 2.1 | Participants

Children with CP and typically developing controls matched to children with CP for age (± 1.5 y) and sex were recruited for the study. Inclusion criteria for children with CP included those who were 5–11 y old, had spasticity and were able to ambulate independently. Inclusion criteria for controls included no history of neurological or motor disorders, and not taking medications that affect musculoskeletal health. The study was approved by the Institutional Review Board at the University of Georgia. Informed consent was obtained from the parent or legal guardian and assent was obtained from the participant, if able, before data collection was initiated.

### 2.2 | Gross motor function

Gross motor function in children with CP was assessed using the Gross Motor Function Classification System (GMFCS), a five-point scale with higher ratings indicating greater motor deficits (Palisano et al., 1997). Briefly, level I indicates an ability to walk indoors and outdoors and climb stairs with no limitations. Level V indicates severe limitations and an inability to ambulate.

### 2.3 | Anthropometrics

Height was measured using a stadiometer (Seca 217; Seca GmbH & Co. KG., Hamburg, Germany) to the nearest 0.1 cm. Body mass was measured using a digital scale (Detecto, 6550, Cardinal Scale, Webb City, MO) to the nearest 0.1 kg.

### 2.4 | LSUT protocol

**FIGURE 1:**
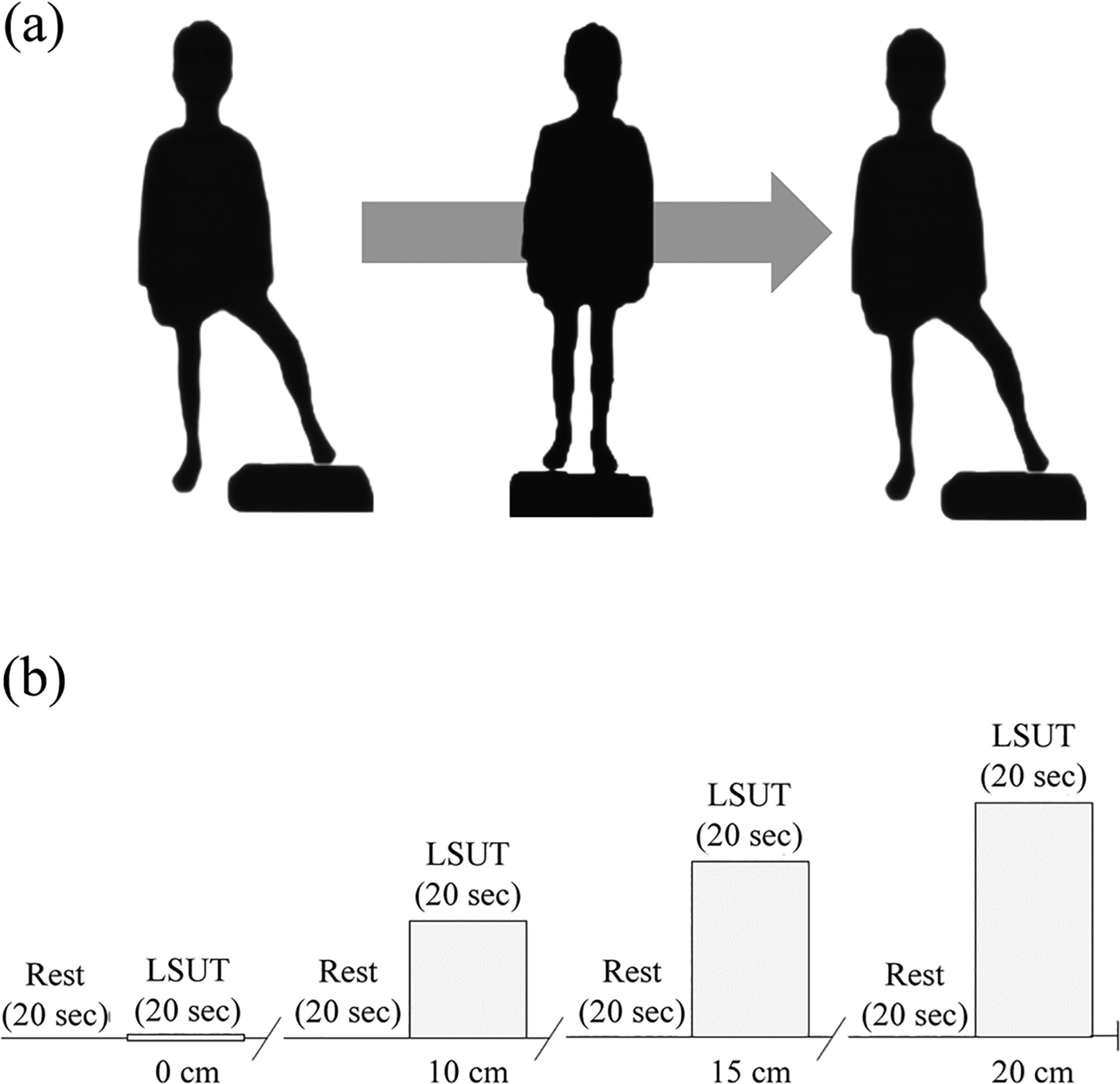
A graphical representation of one successful repetition during the progressive lateral step-up test (LSUT; a). Successful repetitions included a heel strike of the non-tested limb on the floor, and a return to the original position without support. A graphical illustration of the progressive LSUT block paradigm used for the experimental task (b). The LSUT included single trials at 0, 10, 15 and 20 cm step heights. The block paradigm began with 20 seconds of rest. Performance at the 0, 10 and 15 cm step heights was followed by a 30 second setup and 20 second rest period.

The progressive LSUT consisted of four, 20-second trials and a progressive increase in step height for each successive trial (0, 10, 15 and 20 cm, respectively). For the 10, 15 and 20 cm trials, the foot of the more affected lower extremity of children with CP and the non-dominant lower extremity of controls was placed on a step platform and considered the tested limb. Feet were placed shoulder-width apart. Participants were instructed to lift the resting limb, place it next to the tested limb and then return it to its original position. Participants were instructed to perform as many repetitions as possible. Repetitions were successful if the heel of the non-tested limb touched the floor and returned to the original position without support. Every repetition not done independently was counted as an assisted repetition. A graphical representation of one successful LSUT repetition is presented in Figure 1a.

Physical demonstration of optimal performance was provided by test administrators, and participants could perform 1–2 practice bouts prior to each trial. Each trial was preceded by a 20-second rest period, with the child being instructed to stand as still as possible while focusing on an “X” sign located at eye level, 4.5 m away. The single-trial block paradigm is presented in Figure 1b. Performance results of the LSUT were calculated using a weighted, difficulty (i.e., step height)-based scoring system accounting for all repetitions. Repetitions that required assistance were multiplied by 0.1. Step height-adjusted repetitions (repetitions_adj_) were calculated by multiplying repetitions (unassisted + assisted) at 0 cm by 0.5, at 10 cm by 1, at 15 cm by 1.5 and at 20 cm by 2. A composite LSUT score was generated by adding repetitions_adj_ at each step height.

### 2.5 | fNIRS data acquisition

**FIGURE 2:**
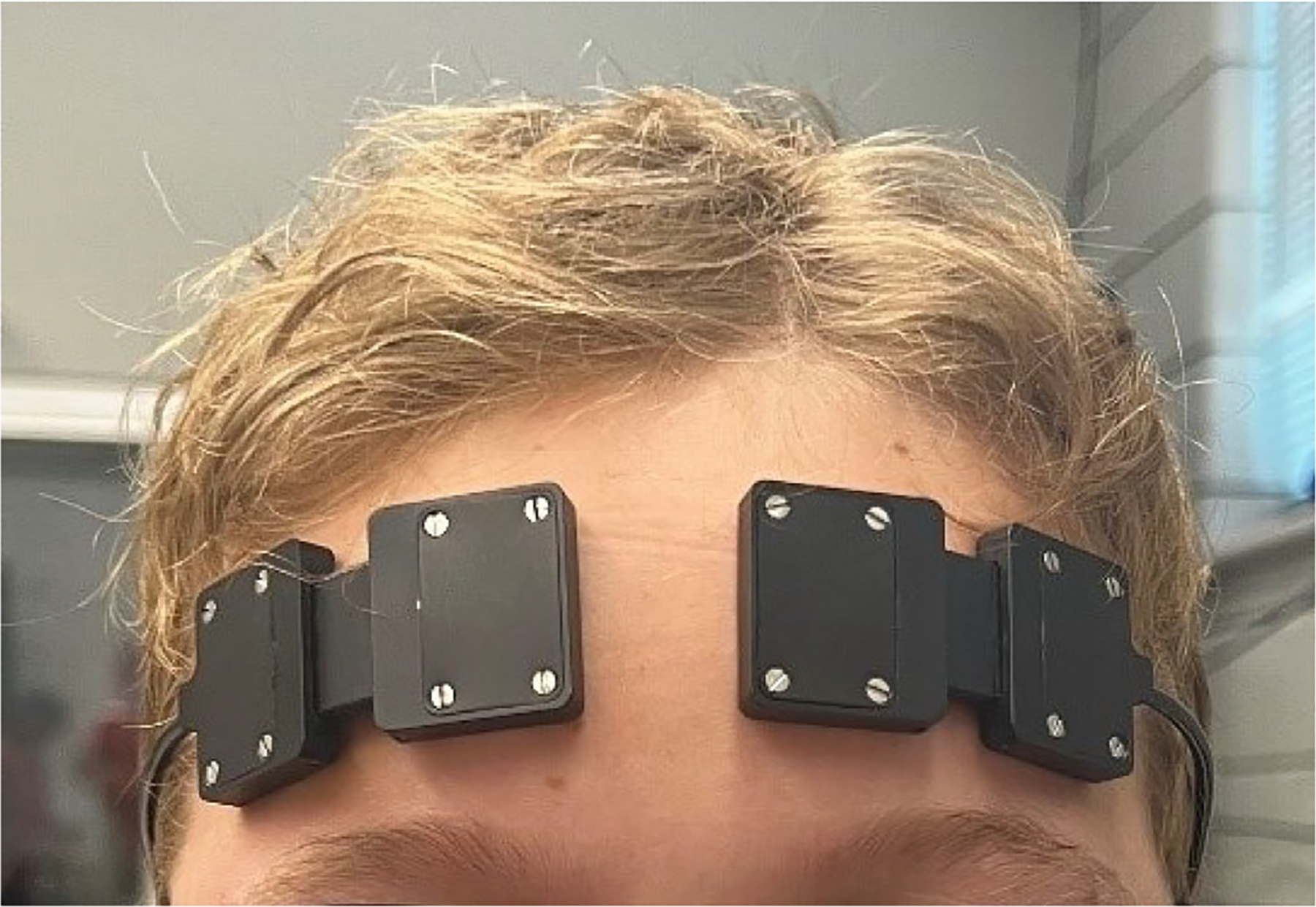
Placement of the functional near-infrared spectroscopy (fNIRS) devices over the participants’ forehead to assess activation of the prefrontal cortex in each hemisphere.

Two portable, continuous wave fNIRS devices (Portalite, Artinis Medical Systems, Einsteinweg, The Netherlands) using two wavelengths (~750 and ~850 nm) were used. Data were sampled at 50 Hz. Each device consisted of three LED optode sources placed at fixed distances of 30, 35 and 40 mm, respectively, from a single optode detector. The optimal source-detector distance has been shown to be age-dependent. While a 15-to-25 mm distance has been recommended for infants 0–2 y (Cai et al., 2021), a 30-to-35 mm distance has been recommended for adults (Li et al., 2011). Considering that children aged 5–11 y were investigated in the present study, the 30 mm channel was selected, consistent with previous work involving children (Eng et al., 2022). Devices were secured to the forehead using double-sided adhesive (Figure 2) over areas corresponding to Brodmann’s areas 9 and 10 (Homan et al., 1987) and covered with black felt to minimize noise. Oxysoft software (v3.2.51, Artinis Medical Systems; Einsteinweg, The Netherlands) was used for data collection. Task events were manually marked.

### 2.6 | fNIRS data processing

Raw fNIRS data were analysed using MATLAB (MathWorks; Natick, MA, United States of America) and the HOMER3 package (Huppert et al., 2009). Signal processing followed recommended practices for fNIRS studies (Yucel et al., 2021). First, raw light voltage intensities were converted to changes in optical density. A principal component analysis (PCA) filter was first applied to the optical density data, using commonly employed parameters (nSV = 0.80 in HOMER3) (Hocke et al., 2018). PCA is a data-driven approach to identify and remove spatially covarying signal components across multiple channels (e.g., large motion artefacts, superficial non-task evoked signal contaminants, low-frequency signal components arising from systemic physiology) that accounted for 80% of the signal variance (Franceschini et al., 2006; Zhang et al., 2005). Further correction for motion artefacts was performed using a SplineSG hybrid technique (Jahani et al., 2018). Baseline shifts were corrected using spline interpolation (Scholkmann et al., 2010), and a robust locally-weighted regression and smoothing Savitzky–Golay filter was applied. Optical density data were filtered using a low-pass filter (0.09 Hz) to account for physiological noise originating from changes in heart rate (Pinti et al., 2018), respiration and Mayer waves (Stefanovska, 2007), and a high-pass filter (0.01 Hz) to account for very low-frequency oscillations (Yucel et al., 2016). Optical densities were converted into relative oxyhemoglobin (HbO) and deoxyhemoglobin (HbR) concentration changes using the modified Beer–Lambert Law, with a constant partial path-length factor of 1. All trials were baseline corrected using the 5 seconds of rest preceding each trial. Relative changes in concentration of HbO (ΔHbO) and HbR (ΔHbR) were obtained for each trial, with ΔHbO and ΔHbR trial averages used for statistical analyses.

### 2.7 | Statistical analysis

Statistical analysis procedures were conducted using RStudio (RStudio Team, Boston, MA). Data were checked for normality by examining skewness and kurtosis and using the Shapiro–Wilk test. Group differences in physical characteristics were assessed using independent t-tests for normally distributed data, and Mann–Whitney *U* tests if data were non-normally distributed. One sample t-tests were used to determine whether the height, body mass and BMI percentiles were different from the 50th age- and sex-based percentiles. Values are presented as mean ± SD unless stated otherwise.

A generalized linear regression model (GLM) was used to assess differences in PFC hemodynamic activation (ΔHbO, ΔHbR) in the PFC across step heights. We used generalized estimating equations (GEE) to account for correlations among repeated measures from the same participant. The GLM with GEE was used to assess the effect of group and step height on repetitions, ΔHbO and ΔHbR during the LSUT. The GLM with GEE was also used to assess the effect of repetitions_adj_ and group on ΔHbO and ΔHbR. Post-hoc comparisons for interactions were performed using the *emmeans* package to compute and contrast estimated marginal means for generalized linear models (Lenth, 2022), with group differences assessed at the mean repetitions_adj_. Post-hoc comparisons for main effects were performed using independent t-tests for normally distributed data and Mann–Whitney *U* tests for non-normally distributed data. A Benjamini-Hochberg procedure was used to correct for multiple comparisons (Benjamini & Hochberg, 1995). Relationships between repetitions_adj_ pooled across step heights and PFC hemodynamic activity were assessed using linear regression analysis. Relationships between repetitions_adj_ and PFC hemodynamic activity at each step height were assessed using *Spearman rho* (*r*_*s*_). Alpha level was set at 0.05. The magnitude of the effects was determined using Cohen’s d (*d*), with 0.2, 0.5 and 0.8 representing small, medium and large effect sizes, respectively (Cohen, 1988).

## 3 | RESULTS

### 3.1 | Participant characteristics

Fourteen ambulatory children with spastic CP (all GMFCS level I) and fourteen typically developing controls participated in the study. There were no group differences in age or physical characteristics (all *p* > 0.05; Table 1). Percentiles for height, body mass and BMI were not different from the 50th age- and sex-based percentiles in either group (all *p* > 0.05).

### 3.2 | Evaluation of LSUT performance

**FIGURE 3:**
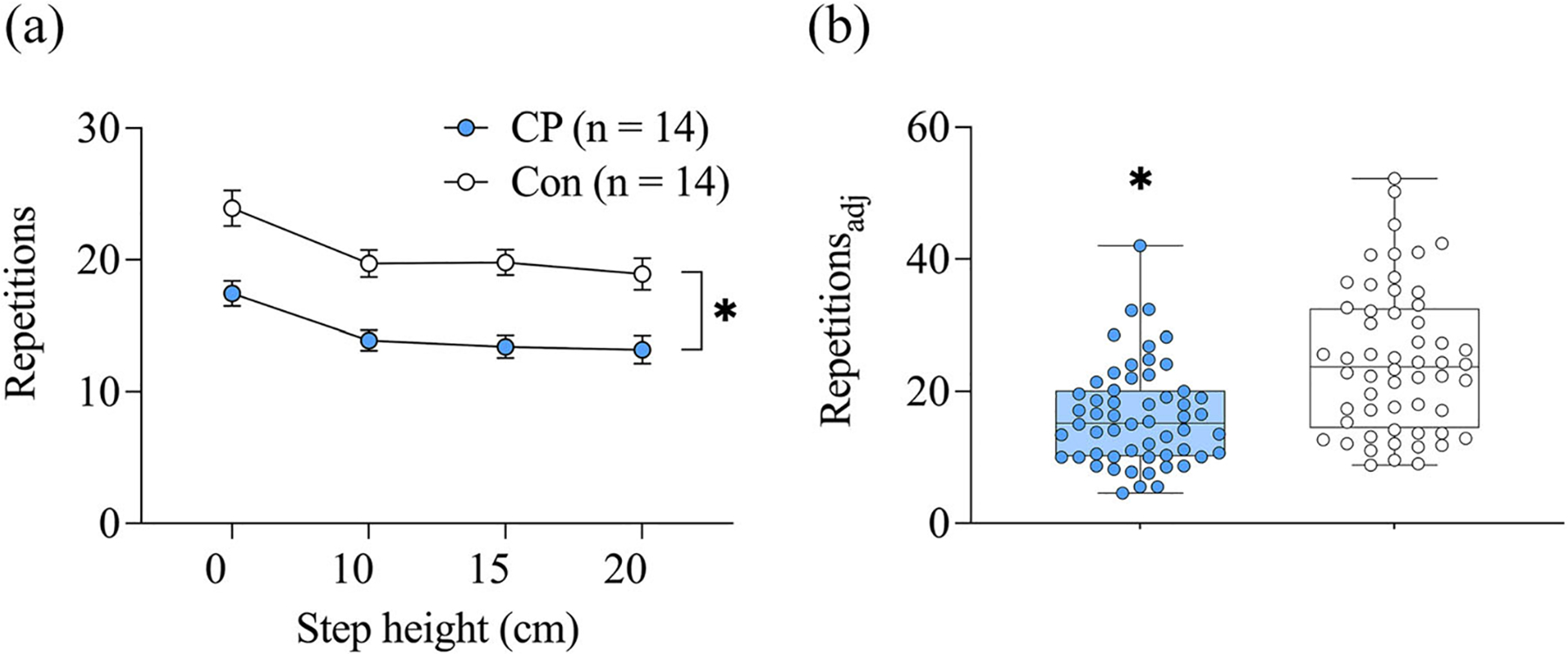
Repetitions (a) and step height-adjusted repetitions (Repetitions_adj_; b) on the progressive lateral step-up test in children with cerebral palsy (CP) and in typically developing children (Con). Values are presented as mean ± SE (a) and as box-and-whisker plots with individual Repetitions_adj_ pooled across all four step heights (b). *Group effect, p < 0.001.

A step height effect was observed with fewer repetitions completed at the 10, 15 and 20 cm step heights relative to the 0 cm height (*p* < 0.001; Figure 3a). A group effect was also observed with children with CP completing fewer repetitions than controls across all step heights (*p* < 0.001; Figure 3a). Children with CP had lower repetitions_adj_ (*d* = 0.90, *p* < 0.001; Figure 3b) and LSUT composite scores (*d* = 2.01, *p* < 0.001) than controls.

### 3.3 | Evaluation of prefrontal cortex hemodynamic activity during the LSUT

**FIGURE 4:**
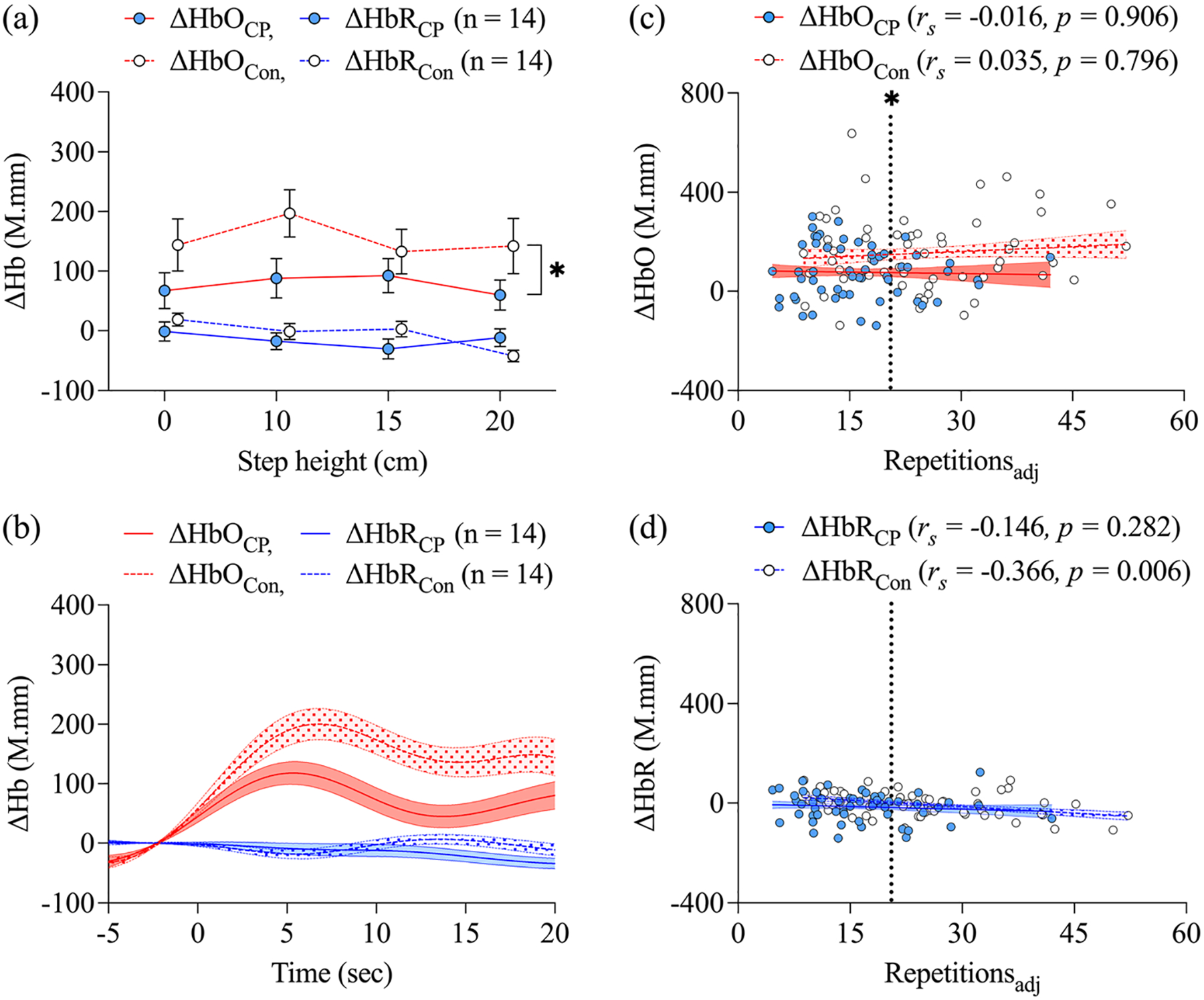
Prefrontal cortex hemodynamic activation reflected by relative changes in concentration of oxyhemoglobin (ΔHbO) and deoxyhemoglobin (ΔHbR) in children with cerebral palsy (CP) and typically developing controls (Con) across step heights of a progressive lateral step-up test (LSUT, a). Group-averaged time series are depicted across the 20-second lateral step-up trial. Time series data were baseline corrected using the mean of the signal from 5 seconds prior (−5 sec) to the start of each LSUT trial (0 sec) (b). Relationships between step height adjusted-repetitions (Repetitions_adj_) and ΔHbO (c) and ΔHbR (d) are illustrated, with dotted vertical lines indicating the Repetitions_adj_ mean for the groups combined (value = 20.5) at which group differences were assessed. Values are presented as mean ± SE. *Group effect.

A statistically significant group effect was observed for ΔHbO, with lower overall ΔHbO noted in children with CP compared to controls (*p* = 0.024; Figure 4a). No step height effect was detected for ΔHbO. While no statistically significant group effect was observed for ΔHbR, a step height effect was noted at the 20 cm step height, with lower ΔHbR observed relative to the 0 cm height (*p* = 0.023). Group-averaged time series data followed the hemodynamic response function pattern (large increase in HbO and smaller decrease in HbR; Figure 4b).

### 3.4 | Relationship between LSUT performance and PFC hemodynamic activity

No significant relationships between LSUT repetitions and PFC hemodynamic activity were observed in children with CP (*r*_*s*_ range = −0.440 to 0.414, all *p* > 0.05). For controls, LSUT repetitions were positively related to ΔHbO (*r*_*s*_ = 0.560, *p* = 0.037) and negatively related to ΔHbR (*r*_*s*_ = −0.618, *p* = 0.019) at the 20 cm step height, but there were no significant relationships at any other step height (*r*_*s*_ range = −0.393 to 0.459, all *p* > 0.05).

A main effect of group was maintained for ΔHbO after accounting for task performance (repetitions_adj_) in the statistical model, with children with CP exhibiting lower ΔHbO than controls overall (*p* = 0.025) and at the mean repetitions_adj_ (*p* = 0.019; Figure 4c). No main effect of repetitions_adj_ on ΔHbO was observed, and relationships between repetitions_adj_ and ΔHbO were not statistically significant for either group, suggesting group differences in ΔHbO were not significantly influenced by task performance.

A main effect of group was also observed for ΔHbR after including repetitions_adj_ in the model (*p* = 0.025; Figure 4d), with lower values observed in children with CP. A main effect of repetitions_adj_ was also noted for ΔHbR (*p* = 0.007), with ΔHbR decreasing with improved task performance, but there was no significant group difference in ΔHbR at the mean repetitions_adj_. No relationship between repetitions_adj_ and ΔHbR was observed in children with CP. An inverse relationship between repetitions_adj_ and ΔHbR was observed in controls (*r*_*s*_ = −0.366, *p* = 0.006), suggesting greater PFC activity was related to better task performance in controls, but not in children with CP.

## 4 | DISCUSSION

This is the first study to assess the effect of a progressive test of lower extremity functional muscle strength on PFC hemodynamic activity in children with CP. Our main finding was that PFC hemodynamic activation was lower in children with CP compared to typically developing controls, suggesting that children with CP process information differently than typically developing children during the progressive LSUT.

The novel observation of decreased ΔHbO in children with CP compared to controls is consistent with previous work reporting decreased PFC hemodynamic activity in children with spastic CP during a robot-assisted walking task (Perpetuini et al., 2022). While another study reported increased PFC hemodynamic activity in children with CP (Surkar et al., 2018), the task involved cognitive shape-matching using the upper extremities and was performed in a seated position. Thus, it placed significantly lower metabolic, postural and attentional demands than the LSUT in the present study and the robot-assisted gait task (Perpetuini et al., 2022). This suggests that cortical resources are directed away from the PFC during physically demanding motor tasks. Reduced PFC oxygenation is also observed during strenuous exercise, with posited mechanisms including a central hemodynamic re-allocation of resources to cortical regions of higher importance for motor output (i.e., transient hypofrontality hypothesis) (Dietrich, 2006; Ogoh & Ainslie, 2009) and a peripheral redistribution of blood to the working muscles (Robertson & Marino, 2016). These hemodynamic effects may be more pronounced in children with CP, given their lower cardiorespiratory capacity (Rimmer, 2001) and disproportionately greater energy expenditure during physical activity (Bell & Davies, 2010) compared to controls.

The lower PFC hemodynamic activation in children with CP may also be attributed to psychological factors. Children with CP experience difficulties with sustained attention (Bottcher et al., 2010) and emotional regulation (Belmonte-Darraz et al., 2021) that could exacerbate the perceived challenge of the progressive LSUT. Additionally, children with CP use greater cortical resources for similar motor output compared to typically developing children, suggesting the amount of cortical resources available decreases as the intensity of activity increases (Short et al., 2020). Notably, task performance (repetitions_adj_) did not influence ΔHbO in either group, with lower ΔHbO consistently noted in children with CP across the range of repetitions_adj_. Task performance similarly impacted ΔHbR in both groups, as suggested by the absence of a group-by-repetitions_adj_ interaction. Interestingly, ΔHbR was significantly lower in children with CP than controls when repetitions_adj_ was included in the model, indicating greater oxygen utilization in the former, but no group differences in ΔHbR were observed at the mean repetitions_adj_. Previous work has demonstrated that the direction of ΔHbR varies as a function of venous oxygenation and volume, making it a less sensitive indicator of cerebral blood flow than ΔHbO (Hoshi et al., 2001). The lack of group differences in ΔHbR could also be due to its lower signal-to-noise ratio compared to ΔHbO (Buxton et al., 1998). Together, these observations suggest altered PFC recruitment patterns during the progressive LSUT in children with CP compared to controls. Greater PFC recruitment by controls positively impacted their task performance at the most challenging 20 cm step height, where higher PFC hemodynamic activity was associated with improved LSUT performance. In contrast, children with CP consistently displayed suppressed PFC recruitment across LSUT step heights compared to controls. The lack of significant associations between PFC hemodynamic activity and task performance in children with CP may indicate either a ceiling effect or that factors other than task performance were driving PFC hemodynamic activity in this group.

The current study has notable strengths. First, the wireless fNIRS devices allowed us to assess PFC hemodynamics during the highly dynamic LSUT in children with CP. Older fNIRS systems used heavy, wired fibre-optic bundles of limited flexibility and short lengths that restricted the ability to assess brain activity during highly mobile tasks (Ferrari & Quaresima, 2012). Second, fNIRS devices were placed over the forehead, where the lack of hair improves signal quality (Orihuela-Espina et al., 2010). Third, groups were matched for age and sex, minimizing the impact of these potential confounders. Fourth, the height, body mass and BMI of the controls were not different from the 50^th^ population percentiles, suggesting they were reasonable representatives of the general population. Finally, the progressive LSUT used in the study incorporated a novel incremental design that allowed for the graded and quantitative evaluation of motor performance in children with CP. Large group differences reflect the high discriminative potential of this test.

The limitations of this study require discussion. First, the fNIRS devices used restricted coverage to a single channel over each PFC hemisphere. Future studies with larger templates should include sensorimotor and premotor areas. The devices also prevented the addition of short separation channels to account for systemic physiological contaminants; however, a PCA filter (nSV = 0.8) was applied to remove signal contaminants before further applying a low-pass (0.09 Hz) and high-pass (0.01 Hz) filter to minimize their influence (Kirilina et al., 2012). Additionally, the block design protocol and use of hemodynamic means for statistical analyses are posited to mitigate the impact of Mayer waves (Izzetoglu & Holtzer, 2020). Second, our small sample size prevented the assessment of potential confounders such as age, sex and type of CP. While the study sample was matched for age and sex, supplementary data for brain injury type and timing that may influence functional outcomes were not collected. Third, measures of peripheral fatigue, central fatigue, cardiorespiratory indicators of task intensity and measures of affect and exercise tolerance that may impact fNIRS outcomes were not assessed and should be included in future studies involving dynamic movements like the LSUT. Finally, our task design prevented us from controlling the number of repetitions completed at each step height, with group differences in LSUT performance possibly influencing the fNIRS signal amplitude. However, a group difference remained for ΔHbO when repetitions_adj_ was included in the statistical model, with lower ΔHbO observed in children with CP at the mean repetitions_adj_. No notable relationship between LSUT performance and ΔHbO was observed and task performance was not related to PFC hemodynamic activity at any step height in children with CP, suggesting that other factors were driving the lower PFC activation observed in children with CP.

## 5 | CONCLUSION

This study provides novel results suggesting PFC hemodynamic activity patterns vary between children with CP and typically developing children, and may be influenced by task-specific physiological and/or psychological demands. Further research is recommended to understand the mechanisms underlying the suppressed PFC hemodynamic activity observed in children with CP.

## Figures and Tables

**TABLE 1:** Physical characteristics in children with cerebral palsy (CP) and in typically developing children (Con).

	CP (n = 14)	Con (n = 14)	*d*	*p*
Age (years)	9.1 ± 2.0	9.0 ± 2.2	0.06	0.88
Sex (M/F)	8/6	8/6	―	―
Height (m)	1.33 ± 0.13	1.35 ± 0.14	0.18	0.63
Height (%)	44 ± 30	61 ± 26	0.58	0.13
Body mass (kg)	32.1 ± 8.5	32.9 ± 12.0	0.07	0.86
Body mass (%)	58 ± 31	57 ± 31	0.02	0.96
BMI	18.1 ± 2.9	17.5 ± 3.4	0.21	0.59
BMI (%)	64 ± 31	55 ± 34	0.26	0.51
Lower limb dominance (L/R)	8/6	2/12	―	―

Values are mean ± SD. BMI, Body mass index. % for height, body mass, and BMI reflect the percentile relative to age- and sex-based norms.

## Data Availability

The data that support the findings of this study are openly available in Figshare at https://doi.org/10.6084/m9.figshare.24251236.

## Abbreviations:

BMI: Body mass index
Con: Typically developing children
CP: Cerebral palsy
EEG: Electroencaphalography
MRI: Magnetic resonance imaging
fNIRS: Functional near-infrared spectroscopy
GEE: General estimating equation
GLM: Generalized linear model
GMFCS: Gross Motor Function Classification System
HbO: Oxyhemoglobin
HbR: Deoxyhemoglobin
LSUT: Lateral step-up test
PCA: Principal component analysis
PFC: Prefrontal cortex
Repetitions_adj_: Step height-adjusted repetitions
